# What Do Biomedical NER and Entity Linking Benchmarks Measure? A Corpus-Centric Diagnostic Framework

**Published:** 2026-05-19

**Authors:** Robert Leaman, Rezarta Islamaj, Zhiyong Lu

**Affiliations:** National Library of Medicine, Bethesda, MD

## Abstract

Biomedical named entity recognition (NER) and entity linking (EL) strongly depend on annotated corpora, but the utility of these resources for benchmarking is often assumed rather than characterized. We present a corpus-centric framework for diagnosing benchmark-relevant properties directly from corpus annotations, concept links, train-test splits, document metadata, and terminology mappings. The framework organizes standardized statistics into five families: (1) scale, density and label distribution, (2) lexical and conceptual structure, (3) train-test overlap, (4) metadata composition, and (5) terminology coverage where applicable. Applying the framework to nine corpora spanning diseases, chemicals, and cell types, we find that corpus properties can differ substantially, even when they address the same apparent task. We find differences in the evaluation signal they provide, the generalization demands they impose, the degree of train–test reuse they permit, and the regions of biomedical literature and concept space they represent. These differences suggest that commonly reported corpus statistics can be insufficient to characterize what biomedical NER and EL benchmarks evaluate. We argue that corpus-centric diagnostics provide a practical framework for analyzing corpora beyond surface descriptors such as corpus size and entity type, for identifying potential transfer risks, and for interpreting the scope of benchmarking conclusions. We release the framework as open-source code^1^ with an interactive dashboard to support reproducing our analyses and characterizing additional corpora.

## 1 Introduction

Extracting structured information from biomedical literature requires systems to identify and link entities (e.g., genes, diseases, chemicals) to standardized identifiers. These grounding tasks—named entity recognition (NER) and entity linking (EL)—remain critical in the large language models (LLM) era to ensure outputs are auditable, comparable, and reusable.

Manually annotated corpora serve both as training data and as evaluation benchmarks (Kim et al., 2003; Collier et al., 2004; Morgan et al., 2008; Lu et al., 2011; Wei et al., 2013; Doğan et al., 2014; Krallinger et al., 2015; Li et al., 2016; Islamaj et al., 2021b,a; Bada et al., 2012; Herrero-Zazo et al., 2013; Wei et al., 2016; Miranda-Escalada et al., 2023). When used as benchmarks, they function as measurement instruments: the key question is not only whether annotations are correct, but what capabilities the benchmark tests and whether conclusions transfer to intended use cases.

This distinction matters because benchmark utility is task- and domain-dependent. A corpus can be carefully annotated yet too narrow, homogeneous, or leaky across splits to support informative evaluation. Biomedical natural language processing (NLP) values rare, specialized, and emerging concepts, making evaluation sensitive to which entities, subdomains, time periods, and document types are represented. Without characterizing the *corpus domain* and the target *application domain*, it is difficult to distinguish generalization issues from corpus-specific artifacts, leakage, or domain mismatch. Benchmark corpora have primarily been compared by size, entity type, or reported system performance. These descriptors do not reflect overlap risks, lexical difficulty, domain bias, or concept coverage.

**Figure 1:**
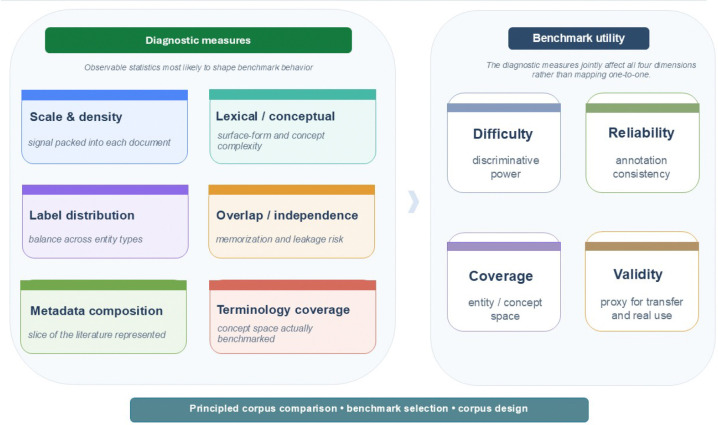
Corpus diagnostic framework. Entity-annotated corpora are converted into a common representation, enabling computation of statistics over annotations, identifiers, and metadata. These statistics characterize scale and density, lexical and conceptual variation, train-test overlap, metadata composition, and terminology coverage, enabling principled comparison of biomedical NER and EL benchmarks.

To address this gap, we introduce a corpus-centric framework that computes standardized statistics directly from annotations, concept links, corpus splits, metadata, and terminologies (Figure 1). These statistics provide a multidimensional analysis for PubMed- and PMC-based NER and EL corpora: density indicates how much evaluation signal is available; lexical and conceptual variation indicate what kinds of generalization are required; overlap reveals leakage risk; metadata composition characterizes the literature represented; and terminology coverage indicates which parts of the concept space are represented.

Applied to nine corpora spanning diseases, chemicals, and cell types, the framework shows that resources similar by task label or size can differ substantially across these signals. Our contributions are: (1) a corpus-as-instrument framing for biomedical NER and EL benchmarks; (2) a practical framework of corpus-centric diagnostics; (3) an analysis showing how structural differences inform evaluation sensitivity, leakage risk, coverage, and transferability; and (4) open-source code and an interactive dashboard for reproducing our results and for analyzing new corpora.

## 2 Related Work

Biomedical entity annotation corpora have proliferated over the years, yet they are routinely treated as benchmarks without any systematic analysis of what they actually measure. Early efforts, such as GENIA (Kim et al., 2003) established large-scale manual mention annotation with fine-grained semantic categories, later simplified for shared tasks such as JNLPBA (Collier et al., 2004). Subsequent corpora introduced entity normalization, typically targeting a single entity type: diseases (NCBI Disease), chemicals (CHEMDNER, BC5CDR), genomic variants (tmVar) and genes (BioCreative challenges) (Doğan et al., 2014; Krallinger et al., 2015; Li et al., 2016; Wei et al., 2013; Morgan et al., 2008). Resources such as CRAFT (Bada et al., 2012) broadened the scope to multi-entity, full-text annotation. More recent corpora—including NLM-Chem, BioRED, and CellLink–have further expanded document and entity coverage, and incorporate richer annotation structures such as relations (Islamaj et al., 2021b, 2024; Rotenberg et al., 2026). Despite being widely used together, these corpora vary considerably in document type, annotation density, normalization support, temporal range, and domain focus—differences that are rarely examined in terms of what each benchmark actually evaluates.

NLP research has shown that dataset properties can distort benchmark interpretation. Work on saturation motivates aggregated benchmarks such as GLUE and SuperGLUE (Wang et al., 2018, 2019); work on artifacts, leakage, and memorization shows that apparent improvements can reflect shortcuts or overlap rather than the intended capability (Gururangan et al., 2018; Liang et al., 2023; Tutubalina et al., 2020); and HELM emphasizes multi-metric evaluation (Liang et al., 2023). Biomedical suites such as BLUE, BLURB, and BigBIO standardize cross-task evaluation (Peng et al., 2019; Gu et al., 2021; Fries et al., 2022), but generally treat corpora as fixed inputs rather than explaining how corpus properties shape validity or transferability.

Annotation quality measures, particularly inter-annotator agreement (IAA), assess consistency but not benchmark scope. Prior work distinguishes agreement across span boundaries, labels, and concept links (Artstein and Poesio, 2008), recommends F1 for span-based tasks lacking a well-defined negative class (Hripcsak and Rothschild, 2005), and notes that disagreement may reflect ambiguity, error, or guideline limitations (Aroyo and Welty, 2015; Uma et al., 2021). High agreement is necessary but not sufficient: simplifying annotation can increase agreement while removing realistic ambiguity (Hovy and Lavid, 2010). Corpus papers often report counts and distributions, but these statistics are rarely organized around evaluation claims. Our framework connects such descriptions to overlap, memorization, domain shift, annotation scope, and terminology coverage.

## 3 Methods

### 3.1 Framework Overview and Representation

Our framework characterizes NER and EL corpora through four stages: conversion to a shared representation, filtering, metric computation, and visualization. Corpora are standardized into documents containing text, optional metadata, and annotations (spans, surface forms, labels, and linked concept identifiers).

This design supports both NER-only and NER+EL PubMed- or PMC-based corpora. NER-only corpora are evaluated on text-, span-, and mention-level statistics, while EL-supported datasets additionally yield concept-level diagnostics. Metrics are computed over configurable corpus bundles, comparison suites, entity scopes, and train/dev/test partitions to enable interpretable comparisons across heterogeneous datasets.

### 3.2 Corpora Analyzed

We apply the framework to nine corpora spanning diverse entity types (e.g., diseases, chemicals, cell types) and document scopes (abstracts, captions, full-text): AnatEM (Pyysalo and Ananiadou, 2014), BC5CDR (Li et al., 2016), BioID (Arighi et al., 2017), CHEMDNER (Krallinger et al., 2015), CRAFT (Bada et al., 2012), CellLink (Rotenberg et al., 2026), JNLPBA (Collier et al., 2004), NCBI-Disease (Doğan et al., 2014), and NLM-Chem (Islamaj et al., 2021b). Where public test data were unavailable or original annotation layers were altered, we utilized the closest documented subset and note these limitations alongside the relevant results.

### 3.3 Diagnostic Statistics

The framework computes corpus statistics across five families to diagnose benchmark properties prior to system evaluation:

- **Scale, Density, and Label Distribution:** We compute total documents, tokens, annotations, and unique mentions/identifiers per document.
- **Lexical and Conceptual Structure:** For normalized corpora, we measure mention ambiguity (the number of distinct label/link pairs mapped to a single surface form) and identifier variation (the number of distinct surface forms mapped to a label/link pair). These distinguish a benchmark’s demand for contextual disambiguation versus its demand for recognizing diverse lexical realizations.
- **Train-Test Overlap:** To assess leakage and memorization risk, we compute Jaccard overlap between train and test splits at four abstraction levels: general token vocabulary, tokens inside entity mentions, exact mention strings, and concept identifiers.
- **Metadata Composition:** We profile the represented literature slice via temporal statistics (publication year ranges and distributions) and journal diversity (unique journals and top-journal concentration). We derive broad topic profiles from article Medical Subject Headings (MeSH) (Lipscomb, 2000) topics where available, falling back to NLM Catalog MeSH journal topics if necessary.
- **Terminology-Aware Coverage:** For diseases and chemicals, we link identifiers to their respective concepts in MeSH, MONDO (Vasilevsky et al., 2026), or ChEBI (Malik et al., 2026); for cell types, we support Cell Ontology (CL) (Tan et al., 2026). We quantify vocabulary coverage by analyzing the distribution of annotations across high-level branches and compute hierarchy depth as a proxy for concept specificity.

### 3.4 Implementation

The framework is implemented as an open-source, YAML-configurable Python pipeline that outputs structured JSON statistics. It includes acquisition specifications for downloading, extracting, converting, caching, and validating expected corpus files. Input support includes BioC XML (Comeau et al., 2013, 2019), PubTator (Wei et al., 2024), BRAT/standoff (Stenetorp et al., 2012), and Knowtator (Ogren, 2006) annotations, with registry-based extension points for additional loaders and metrics. Ontologies in OBO format are directly supported as terminology sources. The accompanying self-contained HTML/JavaScript dashboard^2^ combines scale, overlap, metadata, terminology, and entity-scope views to reproduce our analyses and evaluate new corpora.

## 4 Results

We use the nine corpora to illustrate how corpus-centric diagnostics clarify the evaluation role of a benchmark. The goal is not to rank corpora, but to demonstrate that datasets with similar task labels often function as different measurement instruments: they expose systems to different volumes of evaluation signal, different forms of lexical and conceptual generalization, different leakage risks, and different regions of the biomedical literature and concept space.

### 4.1 Scale, annotation density, lexical and conceptual variation

Table 1 reports statistics for nine heterogeneous biomedical corpora. These measurements show that corpora differ fundamentally in the structural nature of the evaluation signal they provide. Annotation density varies widely across corpora, reflecting differences in document scope, annotation unit, and entity scope. Dense full-text corpora such as NLM-Chem and CRAFT provide many labeled decisions per article, whereas passage-, abstract-, and caption-based corpora distribute fewer decisions across more sampled text units. This raw density is useful because it affects the number of system decisions contributing to an evaluation estimate. However, annotations from the same full-text article are not necessarily independent: repeated mentions, recurring identifiers, and section-specific language can increase decision volume without proportionally increasing lexical, conceptual, or contextual diversity. Density should therefore be interpreted as signal concentration, not as a direct measure of task difficulty or benchmark quality. In this sense, full-text corpora evaluate behavior over long-document contexts and repeated real-world usage, while shorter-unit corpora may provide broader sampling of independent contexts per annotation. Concept-level diagnostics further define what the instrument is calibrated to measure. Variation ranges from 1.48 surface forms per label/link pair in BioID to 3.74 in CellLink, indicating differing demands on lexical generalization.

### 4.2 Train-Test Overlap

**Figure 2:**
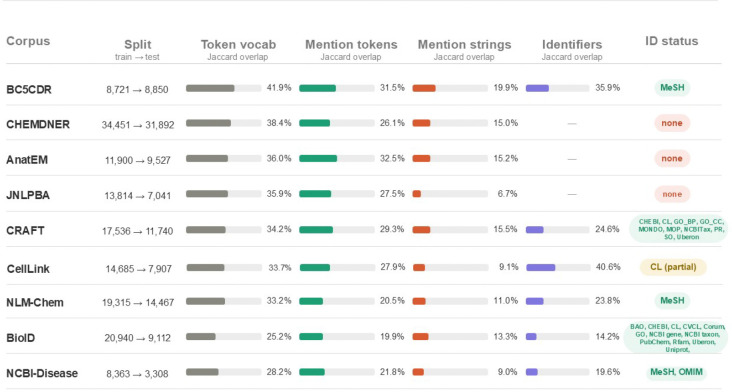
Train-test overlap across nine biomedical corpora. All values are Jaccard similarity (%) between training and test splits. Token vocab: unique vocabulary tokens shared between splits. Mention tokens: unique tokens within entity mention spans. Mention strings: exact entity surface forms shared between splits. Identifiers: concept identifiers shared between splits.

Figure 2 reports train-test Jaccard overlap at four levels: token vocabulary, mention tokens, exact mention strings, and concept identifiers. These levels distinguish increasingly task-specific forms of reuse. Across all corpora, token vocabulary overlap is higher than mention-token overlap, which is, in turn, higher than exact mention string overlap.

The slope of this drop varies and is itself informative. JNLPBA falls from 35.9% token overlap to 6.6% mention-string overlap, indicating that its test split contains relatively little exact entity-form reuse. CellLink presents a different structural pattern: its mention-token overlap (27.9%) is much higher than its exact mention-string overlap (9.1%), while identifier overlap is higher still (40.6%). This profile suggests compositional novelty, where new cell-population names are constructed from familiar component tokens and mapped to familiar concepts. Examples include “CD4+ T cells” (CL:0000624), “resting CD4 memory T cells” (CL:0000897), and “resting NK cells” (CL:0000623), which combine recognizable modifiers with cell-type heads.

Identifier overlap adds a concept-level view unavailable from strings alone. Among normalized corpora, identifier overlap ranges from 14.2% in BioID to 40.6% in CellLink—a range wider than the corresponding mention string overlaps, and with a different ordering. CellLink’s high identifier overlap paired with low string overlap means it tests the ability to map novel text to relatively familiar concepts. Conversely, NCBI-Disease’s low identifier overlap requires systems to generalize to genuinely novel conceptual territory.

### 4.3 Metadata Composition

Temporal coverage differs sharply: CellLink is most recent, spanning 2019–2025; CHEMDNER is nearly a single-year snapshot (97.7% from 2013); CRAFT spans 2001–2007; NCBI-Disease is predominantly pre-2000 (90.8%); and BC5CDR is the broadest, spanning 1968–2016.

The journal distribution shows similar variation in domain. BC5CDR is by far the most venue-diverse: 703 journals, with the top five journals accounting for only 5.0% of the corpus. BioID represents the opposite extreme, with 18 journals total and 83.2% of corpus documents from the top five journals.

The article-topic mappings in Table 2 demonstrate substantial differences in the slice of biomedical literature these corpora represent. NCBI-Disease is strongest in genetics/genomics, CHEMDNER and NLM-Chem emphasize chemistry/materials, BioID is dominated by molecular biology/biochemistry, and CellLink is enriched for general biology and cell/developmental biology.

### 4.4 Terminology-based coverage

**Figure 3:**
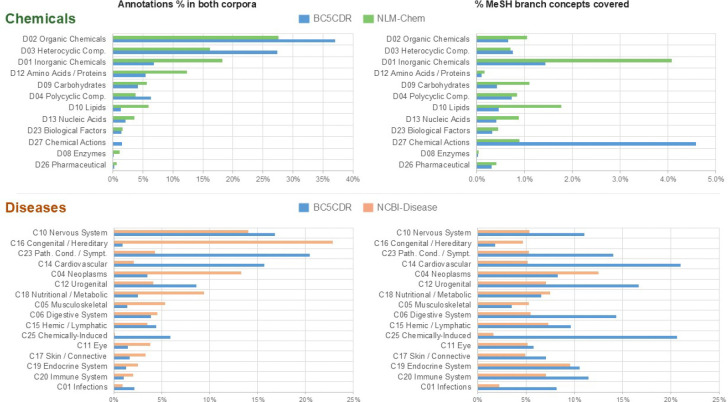
Terminology-based coverage analysis. Left panels: distribution normalized within each corpus’s own annotations. Right panels: distribution normalized within the MeSH tree vocabulary. Top panels: distribution across MeSH chemical branches (D-branches) for BC5CDR and NLM-Chem. Bottom panels: distribution across MeSH disease branches (C-branches) for BC5CDR and NCBI-Disease.

Figure 3 demonstrates that corpora sharing an entity label may still exercise different concept regions of the concept space. The left panels normalize branch counts within each corpus, showing what share of the corpus’s own annotations falls into each high-level terminology branch. The right panels normalize the same branch counts by the size of the corresponding terminology branch, highlighting how the corpus represents different areas of the terminology. Figure 3 focuses on MeSH diseases and chemicals; analogous CL-based statistics for cell-type corpora are available in the dashboard.

For diseases, NCBI-Disease is dominated by Congenital and Hereditary Diseases (C16, 23%) and Neoplasms (C04, 14%), consistent with its genetics origin. BC5CDR instead peaks at Pathological Conditions (C23, 20%) and Cardiovascular Diseases (C14, 17%), consistent with pharmacovigilance as a clinically cross-cutting focus rather than a narrow specialty focus. Chemically-Induced Disorders account for 5.8% of BC5CDR disease annotations but only 0.05% of NCBI-Disease. Thus, while both corpora evaluate “disease” recognition, they measure fundamentally distinct capabilities.

For chemicals, BC5CDR is concentrated in Organic Chemicals (D02, 37%) and Heterocyclic Compounds (D03, 28%), the categories containing most small-molecule drugs. NLM-Chem is more broadly distributed, with higher representation of Inorganic Chemicals (D01, 18%) and Amino Acids/Proteins (D12, 12%).

## 5 Discussion

The central implication of this study is that benchmark results for biomedical NER and EL cannot be interpreted independently of the corpora that produced them. A benchmark corpus is not simply a labeled sample of text: it is a measurement instrument whose structure determines which system capabilities are exercised and limits how far evaluation conclusions can reasonably transfer. Because benchmark utility is task- and domain-dependent, resources that share an entity type label can still function as fundamentally different instruments, evaluating different capabilities, imposing different generalization demands, and representing different regions of biomedical literature and concept space. No single statistic captures this multidimensionality. Instead, benchmark interpretation depends on the interaction of several diagnostic signals, and the nine corpora examined here illustrate how these signals combine in practice.

### Annotation density and label distribution.

Annotation density determines how concentrated evaluation signal is within each sampled unit. Dense full-text corpora such as CRAFT and NLM-Chem yield many linked decisions from relatively few articles, which can improve evaluation sensitivity and expose systems to full-text sectional variation. At the same time, raw density is partly confounded with document length and annotation scope. A large number of annotations from one article may include repeated mentions of the same entities, and therefore may not provide the same independent evidence as the same number of annotations sampled from many distinct passages or documents. Sparse corpora based on abstracts, passages, or figure captions require broader sampling to achieve stable estimates, and they provide more independent contexts per annotation. Each design serves different evaluation purposes: full-text corpora emphasize document-level realism and repeated-use behavior, whereas passage- or abstract-based corpora can emphasize breadth of contexts under fixed annotation budgets.

### Lexical and conceptual variation.

Variation determines whether a corpus tests recognition of repeated forms, alternative expressions for known concepts, or generalization to genuinely new ones. CellLink’s variation of 3.74 surface forms per label-link pair reflects the compositional naming conventions of single-cell biology: novel cell-population names are assembled from familiar modifiers and cell-type heads, so systems must generalize across surface forms while mapping to relatively familiar concepts. BC5CDR’s lower variation is consistent with the pharmacovigilance literature, where drug and disease names tend to be standardized.

### Train-test overlap.

Overlap analysis offers a particularly direct diagnostic for interpreting unexpectedly strong or weak results, consistent with prior work on leakage and memorization (Tutubalina et al., 2020; Liang et al., 2023). The steepness of the token-level overlap drop varies and is in itself informative. CellLink illustrates one diagnostic profile: high identifier overlap (40.6%) combined with much lower mention-string overlap (9.1%) means the benchmark emphasizes compositional surface-form generalization rather than novel concept recognition. NCBI-Disease presents the opposite profile, with low identifier overlap (19.6%) that places genuine demands on concept-level generalization. JNLPBA’s sharp drop from 35.9% token overlap to 6.7% mention-string overlap, combined with the absence of normalization, may partially recontextualize its relatively low performance ceiling [e.g., (Huang et al., 2020)]: systems cannot rely heavily on exact entity-form reuse.

### Metadata composition.

Temporal distributions, journal diversity, and topic profiles describe the slice of literature represented by the corpus. These properties define the domain over which benchmark claims can reasonably apply and determine whether conclusions transfer to corpora drawn from different time periods, venues, or subfields. CHEMDNER’s near-total concentration in 2013 chemistry literature and BioID’s extreme journal concentration (83.2% from five journals) are structural features that may limit the generalizability of results. BC5CDR’s breadth across 703 journals and five decades makes performance claims more domain-general. These metadata properties are rarely reported yet have a bearing on whether performance on a benchmark supports claims about a target deployment setting.

### Terminology coverage.

The contrast between NCBI-Disease and BC5CDR illustrates why terminology-aware analysis matters even when two corpora nominally share a task label. Both annotate disease entities, yet NCBI-Disease is dominated by Congenital and Hereditary Diseases (C16) and Neoplasms (C04), reflecting its genetics origin, while BC5CDR peaks at Pathological Conditions (C23) and Cardiovascular Diseases (C14), reflecting its pharmacovigilance focus. Chemically-Induced Disorders (C25) account for 5.8% of BC5CDR disease annotations but only 0.05% of NCBI-Disease. High performance on one therefore supports only limited claims about transfer to the other, despite the shared entity label. The same logic applies to chemical corpora: BC5CDR’s concentration in Organic Chemicals and Heterocyclic Compounds reflects a small-molecule drug emphasis, while NLM-Chem’s broader distribution across Inorganic Chemicals, Amino Acids, and Lipids reflects the wider biochemical scope of full-text molecular biology literature. Differences in terminology coverage may reflect different practical capabilities even when their reported task names are identical.

### From diagnostics to corpus decisions.

Corpus diagnostics are informative not only retrospectively but before a benchmark is finalized. Before releasing a split, developers can compute mention-string and identifier overlap to detect leakage. While extending a corpus, they can sample documents to fill temporal, journal, topic, or terminology-branch gaps. When selecting a benchmark, researchers can choose corpora whose density, overlap profile, and terminology coverage match the intended deployment claim. When combining corpora, they can verify whether a new source adds concept regions not already represented or mainly duplicates familiar strings and identifiers. The goal, then, is to tie benchmark interpretation to corpus characteristics and intended use, rather than assign a single quality score.

### Limitations.

The framework characterizes corpus structure but does not directly predict downstream system rankings or benchmark saturation. Measured ambiguity conflates true polysemy, underspecified guidelines, and annotation errors; the present framework does not distinguish among these sources. Surface-form variation measures observed diversity rather than the full range of synonymy present in the literature. Terminology analyses depend on the version of the reference ontology used. Finally, topic mappings provide practical, lightweight domain diagnostics rather than definitive subject classifications.

## 6 Conclusion

We presented a corpus-centric framework for diagnosing the benchmark utility of biomedical corpora with entity annotations. The framework organizes standardized statistics over annotations, linked identifiers, corpus splits, document metadata, and terminology mappings into five diagnostic families: (1) scale, density and label distribution, (2) lexical and conceptual structure, (3) train-test overlap, (4) metadata composition, and (5) terminology coverage. Applied to nine biomedical NER and EL corpora, these diagnostics show that corpora for the same apparent task can measure substantially different capabilities.

The main implication is that these statistics provide a practical basis for more complete reporting of biomedical NER and EL benchmarks. Corpus reports should describe annotation density, normalization support, lexical and identifier variation, train-test overlap at multiple abstraction levels, temporal and journal composition, and terminology coverage where applicable. Without these measurements, benchmark results are difficult to interpret. Standardized corpus diagnostics would make evaluation claims more interpretable, clarify what a benchmark measures, and support transparent, reproducible, transfer-aware evaluation.

## Figures and Tables

**Table 1:** Basic statistics for each corpus.

Corpus	Doc. type	Docs	Tokens	E	Total Ann.	Ann/doc	Men./doc	IDs/doc	ID vocab	Ambiguity^a^	Variation^b^

AnatEM	abstracts	1,212	259,510	12	13,701	11.3	7.2	—	—	—	—
BC5CDR	abstracts	1,500	297,019	2	29,271	19.5	9.4	6.9	MeSH	1.02	2.47
BioID	captions	13,697	771,248	8	102,742	7.5	4.9	5.2	mixed^c^	1.35	1.48
CHEMDNER	abstracts	10,000	2,092,491	1	84,331	8.4	4.6	—	—	—	—
CRAFT	full text	97	652,168	11	99,623	1027.0	241.9	149.4	mixed^c^	1.02	2.33
CellLink	passages	2,003	227,490	3	14,731	7.3	6.0	4.1	CL	1.07	3.74
JNLPBA	abstracts	2,404	564,660	5	59,963	24.9	16.5	—	—	—	—
NCBI-Disease	abstracts	793	169,561	1	6,892	8.7	5.1	3.2	MeSH/OMIM	1.01	2.75
NLM-Chem	full text	150	789,532	1	38,339	255.6	54.3	34.1	MeSH	1.02	2.42

a **Ambiguity**: mean label/link pairs per unique mention string. Values near 1.0 indicate most mentions map to a single label/link pair.

b **Variation**: mean surface forms per label/link pair. Reported only for corpora with concept-level identifiers; AnatEM, CHEMDNER, and JNLPBA are excluded (n/a).

c BioID and CRAFT link entity types to many ontology identifier resources.

E = number of annotated entity types; Men./doc = unique mention strings per document; IDs/doc = unique concept identifiers per document. CellLink values reflect the currently released annotated train and development partitions.

**Table 2:** Broad research topic composition of each corpus.

Topic	AnatEM	BC5CDR	BioID	CHEM	CRAFT	CellLink	JNLPBA	NCBI	NLM

Molecular bio. / biochemistry	**16%**	6%	**62%**	15%	21%	12%	**36%**	17%	18%
Chemistry / materials science	8%	15%	—	**29%**	4%	4%	11%	5%	**29%**
Genetics/genomics	5%	—	8%	3%	18%	8%	10%	**28%**	4%
General bio. / anatomy / physiology	14%	14%	6%	18%	**23%**	**22%**	11%	11%	14%
Demographic characteristics	7%	**18%**	—	4%	4%	5%	3%	16%	6%
Cell & developmental biology	7%	1%	10%	5%	9%	17%	12%	4%	6%
Clinical specialties by organ system	8%	11%	—	3%	7%	9%	2%	5%	4%
General / internal medicine	6%	6%	7%	2%	1%	3%	0%	1%	2%
Pharmacology	2%	6%	—	6%	—	—	1%	—	2%
Other	27%	23%	7%	15%	13%	20%	14%	13%	15%

Topics are assigned from article MeSH terms where available; unresolved article terms fall back to NLM Catalog MeSH journal topics and configured journal-name anchors. “Other” aggregates all categories not shown. The largest displayed non-Other entry in each column is shown in bold.

Values represent approximate percentage of corpus articles from each topic area. CHEM = CHEMDNER; NCBI = NCBI-Disease; NLM = NLM-Chem.

## Footnotes

2
https://nlm-dir.github.io/CorpusBenchmarking/dashboard.html
